# Tumor protein Tctp regulates axon development in the embryonic visual system

**DOI:** 10.1242/dev.131060

**Published:** 2016-04-01

**Authors:** Cláudio Gouveia Roque, Hovy Ho-Wai Wong, Julie Qiaojin Lin, Christine E. Holt

**Affiliations:** 1 Department of Physiology, Development and Neuroscience, University of Cambridge, Cambridge CB2 3DY, UK; 2 Doctoral Programme in Experimental Biology and Biomedicine, Center for Neuroscience and Cell Biology, University of Coimbra, Coimbra 3004-517, Portugal

**Keywords:** Tctp, *tpt1*, Neural circuitry assembly, Axon guidance, Retinotectal projection, RNA localisation, Retinal ganglion cell

## Abstract

The transcript encoding translationally controlled tumor protein (Tctp), a molecule associated with aggressive breast cancers, was identified among the most abundant in genome-wide screens of axons, suggesting that Tctp is important in neurons. Here, we tested the role of Tctp in retinal axon development in *Xenopus laevis*. We report that Tctp deficiency results in stunted and splayed retinotectal projections that fail to innervate the optic tectum at the normal developmental time owing to impaired axon extension. Tctp-deficient axons exhibit defects associated with mitochondrial dysfunction and we show that Tctp interacts in the axonal compartment with myeloid cell leukemia 1 (Mcl1), a pro-survival member of the Bcl2 family. Mcl1 knockdown gives rise to similar axon misprojection phenotypes, and we provide evidence that the anti-apoptotic activity of Tctp is necessary for the normal development of the retinotectal projection. These findings suggest that Tctp supports the development of the retinotectal projection via its regulation of pro-survival signalling and axonal mitochondrial homeostasis, and establish a novel and fundamental role for Tctp in vertebrate neural circuitry assembly.

## Abstract (precis)

**Highlighted article:** The cancer-associated protein Tctp controls neural circuitry in *Xenopus* via its regulation of pro-survival signalling and axonal mitochondrial homeostasis.

## INTRODUCTION

Motility and invasiveness are traits central to malignancy and growth cone migration alike. In fact, from the associated changes in adhesion to the build-up of protrusive actin dynamics, or the continuous interaction with the surrounding environment, the initial challenges experienced by a metastatic cancer cell resemble in many ways the obstacles overcome by a navigating growth cone as it progresses through the embryonic brain. Curiously, the four families of guidance cues classically associated with axon guidance – ephrins, semaphorins, netrins and slits (Tessier-Lavigne and Goodman, 1996) have emerged as important regulators of cancer progression, in particular during the phases of primary tumour growth and dissemination (Mehlen et al., 2011; Pasquale, 2010; Tamagnone, 2012), suggesting that common signalling pathways might operate in both contexts. Indeed, frequent mutations and copy number variations were recently discovered in axon guidance genes in tumours derived from patients diagnosed with pancreatic ductal adenocarcinoma (Biankin et al., 2012) and liver fluke-associated cholangiocarcinoma (Ong et al., 2012), and several independent genome-wide screens have found cancer-linked transcripts to be well represented in axonal mRNA populations (Andreassi et al., 2010; Gumy et al., 2011; Zivraj et al., 2010).

Transcripts encoding Tctp (gene symbol: *tpt1*) are ranked among the most enriched in the axonal compartment across diverse embryonic and adult neuronal populations, including retinal ganglion cells (Andreassi et al., 2010; Gumy et al., 2011; Taylor et al., 2009; Zivraj et al., 2010). Tctp is an evolutionarily conserved protein implicated in cell growth (Hsu et al., 2007; Kamath et al., 2003) and is particularly well studied in cancer pathogenesis (Amson et al., 2012; Kaarbo et al., 2013; Tuynder et al., 2002). Initially discovered as an abundant mRNA in untranslated, partially suppressed messenger ribonucleoprotein particles in mouse sarcoma ascites cells (Yenofsky et al., 1982), Tctp was subsequently characterised as a protein that is synthesised at a greatly enhanced rate in growing versus non-growing Ehrlich ascites tumour cells (Benndorf et al., 1988; Bohm et al., 1989). Tctp has since been shown to be involved in cellular functions as diverse as DNA damage (Zhang et al., 2012), cell proliferation (Chen et al., 2007) and allergy responses (MacDonald et al., 1995). In addition, Tctp plays an essential, but still not fully understood, role in development; indeed, loss of *tctp* expression in mice results in increased apoptosis and embryonic lethality (Chen et al., 2007; Susini et al., 2008). Tctp has been shown to interact with the anti-apoptotic oncoproteins myeloid cell leukemia 1 (Mcl1) and Bcl2-like protein 1 (Bcl-X_L_; Bcl2l1) (Liu et al., 2005; Yang et al., 2005; Zhang et al., 2002), and to prevent Bcl2-associated protein X (Bax) homodimerisation in the mitochondrial outer membrane (Susini et al., 2008). Notably, *TCTP* mRNA expression is detected in many areas of the adult human brain (Thiele et al., 2000), and TCTP protein levels are downregulated in the temporal cortex of Alzheimer's disease patients (Kim et al., 2001), suggesting that its expression in the CNS remains important after development.

Here, motivated by the parallels between axon growth and cancer cell invasion, we have investigated the role of cancer-associated Tctp in the context of neural connectivity using *Xenopus laevis* larvae as an *in vivo* model system. We report that Tctp functions as a checkpoint for the normal development of the retinotectal projection. Our results also reveal that mitochondrial function and distribution are affected in axons deficient for Tctp. Finally, we link Tctp to the survival machinery of the axon via its interaction with Mcl1, an anti-apoptotic member of the Bcl2 protein family.

## RESULTS

### Expression of *tctp* in the neural retina

**Fig. 1.**
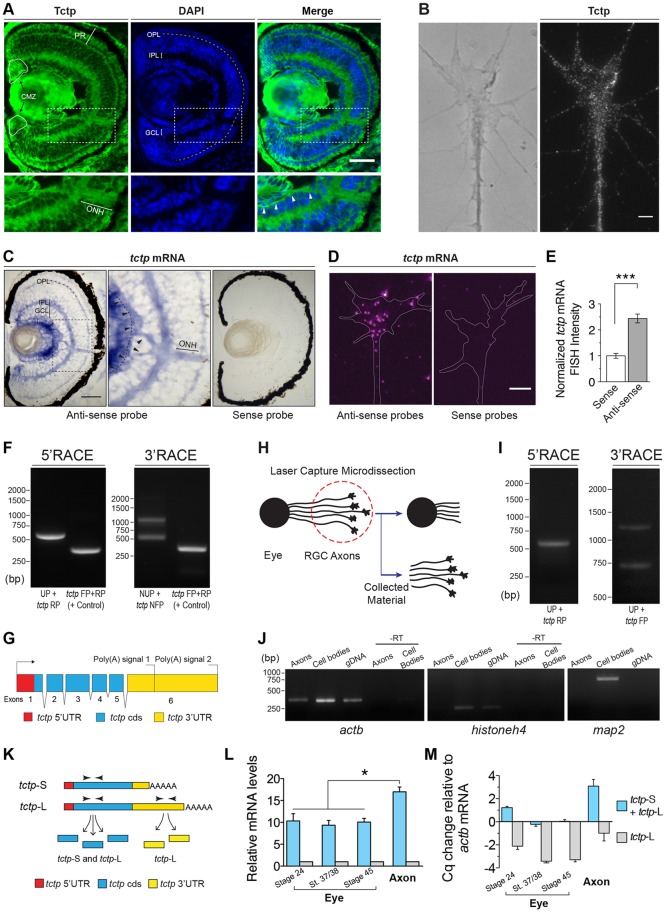
**Expression of *tctp* in the** ***Xenopus*** **neural retina.** (A) Coronal section of stage 43 retina probed with an anti-Tctp antibody and counterstained with DAPI. Arrowheads indicate the optic fibre layer (OFL). The boxed area is enlarged beneath. The dashed contour delineates the outer plexiform layer. (B) Stage 32 eye explants grown *in vitro* for 24* *h were stained with anti-Tctp antibody (left, phase contrast image; right, Tctp antibody staining). Tctp is detected in the axon shaft, central domain and filopodia. (C) *In situ* hybridisation (ISH) detection of *tctp* mRNA expression on coronal sections of stage 43 retinas. Arrowheads indicate the OFL. The boxed area is enlarged in the middle panel. (D,E) Quantitative ISH detection of *tctp* mRNA expression in the RGC axonal and growth cone compartments was performed using stage 32 eye explants grown *in vitro* for 24* *h. Mean±s.e.m.; ***P<0.0001, one-way ANOVA with Bonferroni correction. (F) RACE amplifications of *tctp* mRNAs using retinal RNA extracts. FP, forward primer; NUP, nested universal primer; RP, reverse primer; UP, universal primer. (G) Organisation of the *tctp* gene in *X. laevis*. cds, coding region; poly(A) signal, polyadenylation signal. (H) Schematic of the laser-capture microdissection procedure used to collect RGC axonal extracts. (I) RACE amplifications of *tctp* mRNAs using laser-captured axonal extracts. (J) Purity assessment of laser-captured material by RT-PCR. –RT, RNA samples not reverse transcribed. (K) RT-qPCR experimental design. (L,M) Axonal and whole-eye content of *tctp* mRNAs were analysed by RT-qPCR and normalised to *actb* expression. In L, data are plotted as ‘*tctp*-S+*tctp*-L’ to ‘*tctp*-L’ expression ratios (*P=0.0175, one-way ANOVA), whereas in M the quantification cycle (Cq) difference relative to *actb* is shown. Scale bars: 50 μm in A,C; 5 μm in B,D. CMZ, ciliary marginal zone; GCL, ganglion cell layer; IPL/OPL, inner/outer plexiform layer; ONH, optic nerve head; PR, photoreceptor layer.

Immunohistochemistry using an antibody raised against the *X. laevis* protein revealed that Tctp is broadly present in the retina, including the ganglion cell layer (GCL) and the optic fibre layer (OFL) (Fig. 1A) (Bazile et al., 2009). A strong positive signal is also evident in the optic nerve head (ONH) (Fig. 1A), where retinal ganglion cell (RGC) axons collect to exit the eye, and in RGC axons and growth cones *in vitro* (Fig. 1B). *In situ* hybridisation (ISH) showed a similarly broad expression in the retina that was surprisingly robust in the OFL and ONH, indicating the presence of *tctp* mRNA in retinal axons *in vivo* (Fig. 1C; Fig. S1A,B). This was confirmed by fluorescent ISH on retinal axons and growth cones *in vitro* (Fig. 1D,E; Fig. S1C). The inner and outer plexiform layers (IPL and OPL, respectively) were also positive for Tctp protein and mRNA, suggestive of widespread localisation in the neurites of retinal neurons (Fig. 1A,C). Apart from the retinal neuropil, *tctp* expression was observed in the photoreceptor layer and the ciliary marginal zone (CMZ), a well-characterised retinal neurogenic niche (Fig. 1A,C).

The human *TCTP* gene is transcribed into two distinct mRNA variants that differ only in the length of their 3′ untranslated regions (UTRs) (Thiele et al., 2000). As most mRNA regulatory elements are situated within the 3′UTRs (Martin and Ephrussi, 2009), we investigated whether *tctp* is regulated in an analogous manner in the *X. laevis* retina. Using rapid amplification of 5′ and 3′ cDNA ends (5′ and 3′ RACE), two 3′UTR variants of *tctp* were obtained from eye RNA extracts, comprising a short (*tctp*-S, 210 bases) isoform and a longer (*tctp*-L, 607 bases) version, overlapping in its entirety the short form and possessing a unique stretch at its 3′ end (Fig. 1F). Similar to human, the exon specifying the 3′UTR in *X. laevis* contains two alternative polyadenylation signals, resulting in transcripts with 3′UTRs of different length but encoding the same protein (Fig. 1G). A single 5′ end was identified and, as described in human, sequencing it in its entirety revealed the existence of a 5′-terminal oligopyrimidine (TOP) motif previously not annotated in *X. laevis* (Fig. S1E).

Differential processing at alternative polyadenylation sites is known to be physiologically regulated during development or by pathological events such as cancer, and can affect the localisation and translational properties of the mRNA (Di Giammartino et al., 2011). For example, the longer karyopherin (importin) beta 1 transcript, equally arising from alternative polyadenylation, harbours a signal that enables axonal localisation (Perry et al., 2012). We thus explored whether *tctp* localisation in RGC axons is governed in a similar manner. We used laser-capture microdissection (LCM) to harvest axonal extracts (Zivraj et al., 2010) (Fig. 1H; Fig. S1D). To determine the purity of our pool of axonal mRNAs, we tested for the presence of mRNAs encoding nuclear proteins, such as histone H4 (*hist1h4a*), and for transcripts described in dendrites but not in axons, such as microtubule-associated protein-2 (*map2*). No such amplification products were detected by reverse transcription PCR (RT-PCR) (Fig. 1J). By contrast, *actb* (which encodes β-actin), a transcript previously identified in axons and growth cones (Bassell et al., 1998; Leung et al., 2006), was readily amplified (Fig. 1J). Significantly, *tctp* sequence reads from 5′ and 3′ RACE reactions using RGC axonal extracts were identical to those obtained from whole-eye preparations, implying that both isoforms localise in these axons (Fig. 1I).

We next employed quantitative RT-PCR (RT-qPCR) to complement our analysis. We designed two sets of primers: one directed to a segment of the *tctp* protein-coding region, thus allowing for an expression readout of both mRNA variants, and a second pair targeting part of the unique region of *tctp*-L (Fig. 1K). In whole-eye extracts, an approximately constant 9:1 *tctp*-S to *tctp*-L ratio was obtained at all developmental stages examined. Interestingly, there was a ∼16:1 *tctp*-S to *tctp*-L ratio in axonal extracts, indicating that the *tctp*-S variant is locally enriched in the axonal compartment (Fig. 1L). Moreover, we detected a near tenfold (ΔCq*_tctp_*_:*actb*_=3.1) enrichment over *actb* mRNA, a known axonally enriched mRNA, confirming *tctp* as a highly abundant axonal transcript (Fig. 1M).

### Tctp is required to establish correct axonal projections *in vivo*

**Fig. 2.**
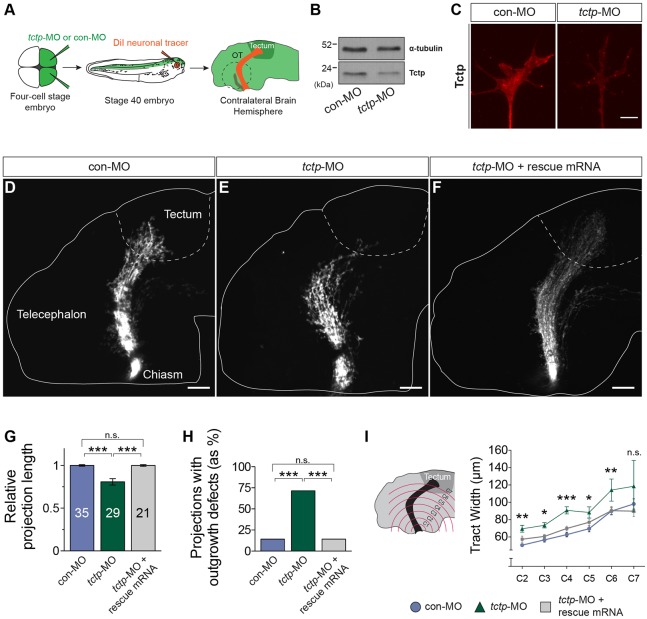
**Tctp is required to establish correct retinotectal projections *in vivo*.** (A) Experimental outline. OT, optic tract. Dashed line encircles the contralateral, DiI-filled eye. (B) *tctp*-MO leads to a specific knockdown in Tctp protein levels in the CNS, as evaluated by western blot analysis of stage 37/38 embryos using an anti-Tctp antibody. (C) Representative growth cones from control morpholino (con-MO)-injected and *tctp*-MO-injected embryos stained for Tctp. (D-F) DiI-filled retinotectal projections in MO-injected stage 40 embryos. Dashed lines approximate the boundary of the optic tectum, where RGC axons terminate. Injection of MO-resistant *tctp* mRNA (rescue mRNA) rescued the development of the retinotectal projection. (G) Relative projection lengths in the various MO-injected backgrounds. Mean±s.e.m.; *n*, number of brains analysed; ***P<0.0001, Kruskal–Wallis test. (H) Number of embryos displaying axon extension defects. con-MO versus *tctp*-MO, *P*<0.0001; *tctp*-MO versus *tctp*-MO+rescue mRNA, *P*=0.0002; Fisher's exact test; performed on number of observations but plotted as percentages. (I) Mean (±s.e.m.) optic tract widths. con-MO versus *tctp*-MO, **P<0.01 (C2), *P<0.05 (C3), ***P<0.0001 (C4), *P<0.05 (C5), **P<0.05 (C6), two-way ANOVA with Bonferroni correction (for details of statistics see Fig. S2F). C2-7 denote imaginary, evenly spaced hemi-circumferences centred on the optic chiasm. Scale bars: 5 μm in C; 100 μm in D-F. n.s., not significant.

We next assessed whether Tctp plays a role in retinal axon guidance. To inhibit *tctp* mRNA translation *in vivo*, we used an antisense morpholino oligonucleotide (MO) directed against the start site of *tctp* mRNA (*tctp*-MO), which was delivered at the four-cell stage by injection into both dorsal blastomeres (Fig. 2A). In doing so, we targeted both *tctp*-S and *tctp*-L transcripts throughout the CNS. Western blot analysis validated the efficient knockdown of Tctp levels (∼50% in brain and eye lysates; *P=0.041, unpaired *t*-test) (Fig. 2B). Similarly, we observed a 40-60% decrease in Tctp protein expression in RGC growth cones from *tctp*-MO-injected embryos (***P<0.0001, Mann–Whitney test), demonstrating that the axonal pool of Tctp is targeted by this approach (Fig. 2C). At the MO dosage used, Tctp morphants appeared morphologically normal, with no overt delays in development, although most individuals showed small decreases in eye size (an average of 10%; **P=0.0063, unpaired *t*-test; Fig. S2A-D). Of note, we titrated an MO dosage capable of achieving an expression knockdown comparable in magnitude to that of *tctp*^+/−^ mice, which are reported to be viable and fertile, unlike *tctp*^−/−^ pups (Chen et al., 2007; Susini et al., 2008).

We analysed RGC axon trajectories by anterograde lipophilic dye (DiI) labelling at stage 40 (∼3-day-old larvae), when the pioneer population of axons have completed their growth through the optic tract and arrived in the optic tectum (Holt and Harris, 1983). Whereas control projections consistently coursed a normal trajectory and had reached the target region by this stage (Fig. 2D), most age-matched Tctp morphants exhibited significantly shorter projections that failed to enter the optic tectum (Fig. 2E,G,H). Additionally, instead of forming the compact axonal bundle typical of normal projections, RGC axons in Tctp morphants grew in a dispersed fashion, straying inappropriately into territories in the diencephalon and telencephalon. Indeed, the optic tract in Tctp morphants was on average ∼21 μm wider than in controls (Fig. 2E,I). Restoring the levels of Tctp with an MO-resistant *tctp* mRNA in *tctp*-MO-injected embryos completely rescued the development of the retinotectal projection both in terms of tract length and tract width, demonstrating that the phenotypes are specific to the loss of Tctp function (Fig. 2F-I; Fig. S2E,F). Collectively, these data demonstrate that Tctp is necessary for the accurate and timely development of the retinotectal projection.

### Tctp promotes axon extension *in vivo*

**Fig. 3.**
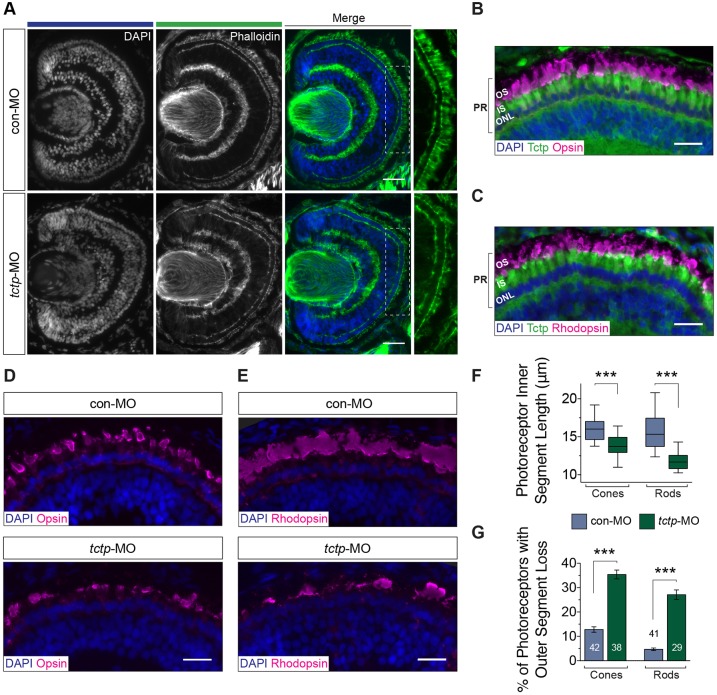
**Tctp is not necessary for the timely development of the eye.** (A) Representative stage 43 control and Tctp-depleted retinas stained with phalloidin and DAPI. The boxed areas are shown at a higher magnification to the right. (B,C) Immunohistochemistry analysis of the photoreceptor layer in stage 43 wild-type retinas probed with anti-Tctp and anti-opsin or anti-rhodopsin antibodies, and counterstained with DAPI. IS, photoreceptor inner segment; ONL, outer nuclear layer; OS, photoreceptor outer segment; PR, photoreceptor. (D,E) Representative micrographs of the photoreceptor layer in stage 43 control or Tctp morphant retinas probed with anti-opsin or anti-rhodopsin antibodies, and counterstained with DAPI. (F) Average inner segment lengths in control and Tctp morphant retinas. ***P<0.0001, unpaired *t*-test; box plot whiskers denote 5th-95th percentile. (G) Proportion of photoreceptors showing a complete loss of the outer segment in control and Tctp morphant retinas. *n*, number of photoreceptor layers analysed; ***P<0.0001, unpaired *t*-test. Scale bars: 50 μm in A; 25 μm in B-E.

The shortened axon projection phenotype in Tctp morphants could arise from a general delay in eye development or a decrease in the rate of RGC axon extension. To distinguish between these possibilities, we first examined the histology of the retina. Overall, although some disorder in the neuropil and an increase in cell death in the GCL were noted, the gross morphology and stratification of the retina appeared unaffected in Tctp morphants, suggesting no major delay in development (Fig. 3A; Fig. S3A-D).

Marked alterations were evident, however, in the photoreceptor layer of Tctp morphants (Fig. 3A). Prompted by the possibility that this defect might provide insight into Tctp action, we further evaluated the photoreceptor phenotype. Briefly, the photoreceptor outer segment is an apical structure densely packed with discs of folded membranes containing light-sensitive photopigments (opsins), whereas the inner segment, which lies between the outer segment and the nuclear layer, is dedicated to sustaining the energy and protein synthesis needs of the photoreceptor (Wright et al., 2010). First, using opsin markers and a nuclear stain, we pinpointed the localisation of Tctp to the mitochondria-rich inner segments of both cone and rod photoreceptors (Fig. 3B,C). Our subsequent analysis revealed that photoreceptors in Tctp morphant retinas have shorter inner segments (cones, 13.7 μm versus 16.1 μm in controls; rods, 11.8 μm versus 15.8 μm in controls), and showed a complete loss of the outer segment in a significant proportion of cones (35% versus 13% in controls) and rods (27% versus 5% in controls) (Fig. 3D-G). Collectively, these data indicate that although Tctp is not essential for the timely development of the retina, it lends an unexpected contribution to photoreceptor maintenance.

**Fig. 4.**
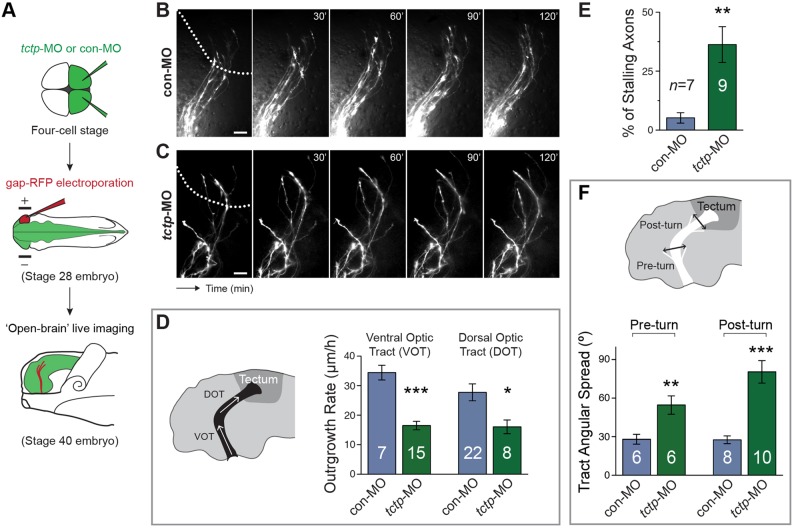
**Tctp deficiency impairs axon extension *in vivo*.** (A) Schematic of the experiment. con-MO-injected or *tctp*-MO-injected stage 28 embryos were electroporated with gap-RFP to label retinal axons and allowed to develop until stage 40 before *in vivo* brain imaging. (B,C) Representative time-lapse images of gap-RFP-labelled control (top) and Tctp-depleted (bottom) RGC axons coursing through the optic tract. Dotted lines approximate the boundary of the optic tectum. (D) Average extension rates measured from time-lapse recordings of RGC axons coursing through the ventral optic tract (VOT) and dorsal optic tract (DOT) in controls and Tctp morphants. VOT, ***P<0.0001; DOT, *P<0.0273. (E) Percentage of axons with stalled progression in the control and morphant backgrounds. **P<0.0035. (F) Retinotectal projection angular spreads in controls and Tctp morphants. Pre-turn, **P<0.0082; post-turn, ***P<0.0001. (D-F) Mean±s.e.m.; *n*, number of axons (D) or embryos (E,F) analysed; unpaired *t*-test. Scale bars: 25 μm.

To measure directly the rate of axon growth *in vivo*, we made time-lapse movies of control and morphant axons using eye-targeted electroporation to deliver gap-RFP (a membrane-targeted version of RFP) (Fig. 4A). Overall, Tctp-depleted axons were significantly slower than control axons, advancing through the optic tract at about half the speed (ventral optic tract, 16.5 μm/h versus 34.4 μm/h in controls; dorsal optic tract, 16.1 μm/h versus 27.8 μm/h in controls) (Fig. 4B-D). In addition, 40% of the morphant axons analysed (33 of 82 axons) stalled along the optic tract, a significantly higher proportion than in control samples (our analysis parameters classified ∼6% of control axons as ‘stalled’; Fig. 4E). As suggested by the fixed DiI samples, time-lapse imaging confirmed that axonal growth in Tctp morphants was dispersed and erratic, which translated into significantly wider projections relative to controls (Fig. 4F). Lastly, we tested whether the tortuous trajectories associated with defective pathfinding could account for the shortened axon tract phenotype detected in Tctp morphants by including only normally projecting axons in our analysis. We found that normally projecting Tctp-depleted axons still extended through the optic tract at significantly slower average rates than controls (Fig. S3E). Collectively, these findings strongly indicate that Tctp regulates retinal axon growth.

### The retinotectal projection develops unerringly in Tctp-deficient brains

**Fig. 5.**
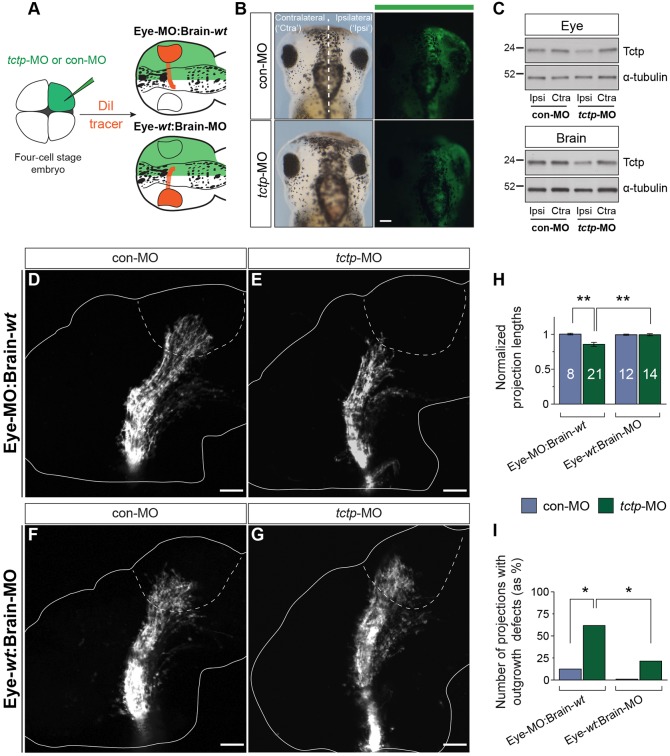
**The retinotectal projection develops unerringly in Tctp-deficient brains.** (A) Experimental outline illustrating the two experimental scenarios created to investigate the contribution of extracellular Tctp to the optic tract pathway substrate. (B) Dorsal view of embryos microinjected unilaterally with fluorescein-tagged con-MO or *tctp*-MO. (C) Unilateral *tctp*-MO injection leads to a targeted knockdown in Tctp expression in half of the CNS, as shown by immunoblot analysis of eye or brain lysates. The ‘ipsilateral’ label refers to the MO-injected half of the embryo; the uninjected half is designated ‘contralateral’. (D-G) DiI-filled stage 40 retinotectal projections. Dashed lines approximate the boundary of the optic tectum. (H) Relative projection lengths. Mean±s.e.m.; *n*, number of brains analysed; **P=0.0002, Kruskal–Wallis and Dunn's multiple comparison test (for details of statistics see Fig. S2F). (I) Number of brains displaying axon extension defects. Eye-MO:Brain-*wt* backgrounds, *P=0.0352; *tctp*-MO backgrounds, *P=0.0364; Fisher's exact test; analyses performed on frequencies but plotted as percentage. Scale bars: 100 μm.

Tctp exhibits immunoglobulin E-dependent histamine-releasing activity and other cytokine-like extracellular functions (Kim et al., 2013; MacDonald et al., 1995). It could therefore work in the embryonic environment to promote axon development. To address this possibility, we injected MOs into only one of the first two dorsal blastomeres, leading to embryos in which one half of the CNS is depleted in Tctp and the other is wild type (Fig. 5A-C). Because RGC axons cross the midline at the optic chiasm and project contralaterally, this strategy enabled us to test the contribution of the optic tract pathway substrate. Embryos injected with control MO (con-MO) consistently developed normal projections in both backgrounds, verifying the suitability of the strategy (Fig. 5D,F). Significantly, Tctp-depleted retinal axons navigating into the contralateral normal (*tctp*-MO-free) hemisphere (Eye-MO:Brain-*wt*) exhibited the same range of phenotypes as observed in global Tctp morphants (Fig. 5E). By contrast, normal RGC axons projecting into the contralateral Tctp-depleted (*tctp*-MO-injected) side of the brain (Eye-*wt*:Brain-MO) showed no defects (Fig. 5G-I; Fig. S2G). Collectively, these findings show that the retinotectal projection can develop unerringly through a Tctp-depleted optic tract neuroepithelium, and indicate that the axonal phenotype of morphant retinal axons in the optic pathway is independent of Tctp acting extracellularly.

### Tctp knockdown compromises axonal mitochondrial function

To begin to investigate the mechanism of how Tctp regulates axon growth, we focused on mitochondria. Tctp is documented as part of the mitochondrial proteome (Fountoulakis et al., 2002; Rezaul et al., 2005), and its expression in the brain is downregulated in pathologies associated with mitochondrial abnormalities, such as Alzheimer's disease and Down syndrome (Kim et al., 2001; Nunnari and Suomalainen, 2012; Pagano and Castello, 2012). Furthermore, photoreceptor degeneration is frequently characterised by bioenergetic decline (Wright et al., 2010), and mitochondrial dysfunction is reported in a number of retinal diseases, including photoreceptor-specific age-related macular degeneration (Alexander et al., 2000; Delettre et al., 2000; Wallace et al., 1988; Wright et al., 2010).

**Fig. 6.**
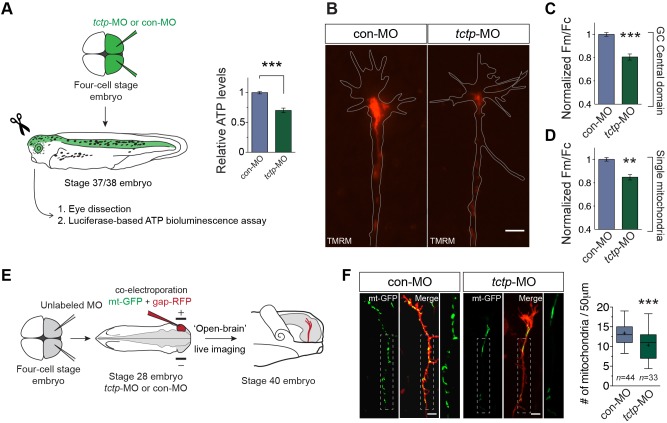
**Compromised mitochondrial homeostasis in axons deficient for Tctp.** (A) Relative ATP levels per retina normalised to total protein. Mean±s.e.m.; *n*=17 per condition, ***P<0.0001, unpaired *t*-test. (B) Representative RGC growth cones loaded with TMRM from con-MO-injected and *tctp*-MO-injected embryos. (C) Quantification of TMRM fluorescence intensity in the mitochondria-rich growth cone (GC) central domain. Mean±s.e.m.; ***P=0.0002, Mann–Whitney test. (D) Quantification of TMRM fluorescence intensity of individual mitochondria along the axonal compartment. Mean±s.e.m.; **P<0.0012, Mann–Whitney test. Up to ten replicate experiments were performed per condition, totalling 427 growth cones and 4918 single mitochondria analysed. (E) Schematic of the approach used to examine mitochondrial density in RGC axons *in vivo*. (F) Micrographs of RGC axons co-labelled with mt-GFP and gap-RFP, plus quantification of axonal mitochondrial density. *n*, number of axons analysed; ***P=0.0002, unpaired *t*-test. In box plots, whiskers cover 5th-95th percentile and ‘+’ indicates the mean. Boxed areas in images are enlarged to the right. Scale bars: 5 μm.

First, we investigated whether the overall metabolic status was changed in Tctp morphant retinas using a bioluminescence ATP assay (Agathocleous et al., 2012). Remarkably, the energy content in Tctp-depleted retinas was found to be, on average, 30% lower than in controls (Fig. 6A). We next measured the mitochondrial membrane potential (ΔΨ_m_), a cardinal indicator of mitochondrial function, in retinal explant cultures. Notably, in Tctp morphants, we found a significantly lower accumulation of the cationic fluorescent probe tetramethylrhodamine methyl ester (TMRM) in the mitochondria-rich growth cone central domain (reduced by ∼20% relative to control), indicating ΔΨ_m_ depolarisation. Analysis of individual mitochondria distributed throughout the axonal compartment showed a comparable ΔΨ_m_ reduction in Tctp morphants (Fig. 6B-D). A significant decrease in the number of axonal mitochondria was also noted in these experiments, which was confirmed *in vitro* with a mitochondrial stain (∼30% fewer mitochondria) and *in vivo* by co-labelling RGC axons with mitochondrion-targeted GFP (mt-GFP) and gap-RFP (∼23% fewer mitochondria) (Fig. 6E,F; Fig. S4A). The average length of axonal mitochondria did not differ from control (Fig. S4B). Collectively, these results show a significant reduction in mitochondrial density in Tctp-depleted axons, as well as a decrease in axonal mitochondrial function and global energy levels.

**Fig. 7.**
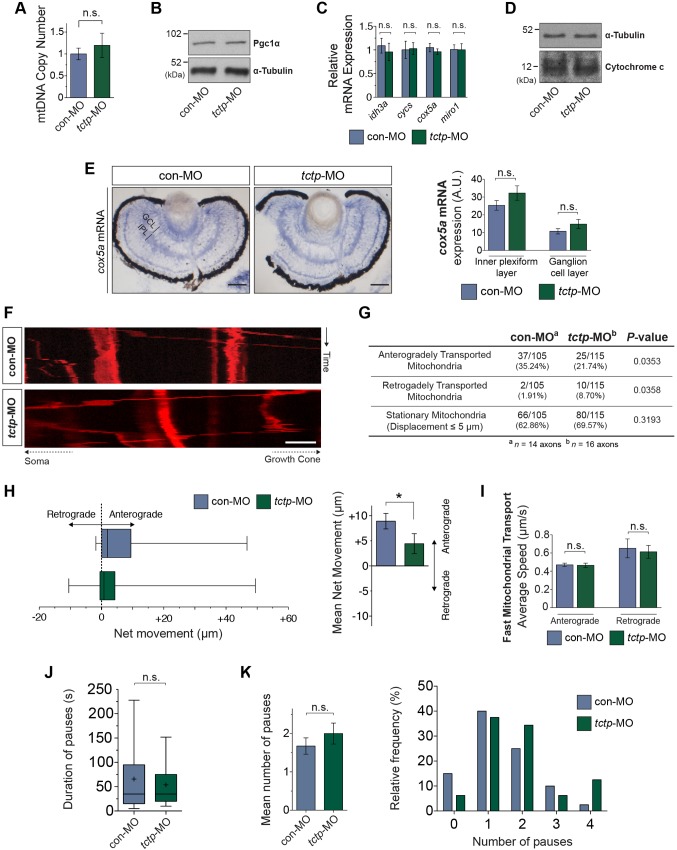
**Altered mitochondrial dynamics in Tctp-depleted axons.** (A) Ratio of mitochondrial to nuclear DNA determined by qPCR in control and Tctp-depleted retinas. Mean±95% confidence interval; *n*=7 paired retinas per condition; *P*=0.23, Mann–Whitney test. (B) Tctp morphants show unaltered Pgc1α expression levels in the CNS as evaluated by western blot analysis of stage 37/38 embryos using an anti-Pgc1α antibody. *n*=3 independent samples; *P*=0.5955, unpaired *t*-test. (C) Tctp morphants show unaltered expression of mitochondria-related genes as assessed by RT-qPCR using eye RNA extracts. Mean±95% confidence interval; *n*=9 retinas per condition, Mann–Whitney test. (D) Tctp morphants show unaltered cytochrome c expression levels in the CNS, as evaluated by western blot analysis of stage 37/38 embryos using an anti-cytochrome c antibody. *n*=3 independent samples; *P*=0.5989, unpaired *t*-test. (E) Control and Tctp-depleted RGCs have similar levels of *cox5a* expression. Mean±s.e.m.; *n*=∼20. GCL, *P*=0.2026; IPL, *P*=0.2668; Mann–Whitney test. (F) Representative kymographs (time-space plots) of MitoTracker-labelled RGC axonal mitochondria in control and Tctp morphant backgrounds. The vertical and horizontal axes represent time and spatial position, respectively (e.g. a vertical line indicates a stationary mitochondrion). (G) Summary of changes in axonal mitochondrial dynamics (statistical significance determined using Fisher's exact test). (H) Relative mitochondrial motility and mean net movement in control and Tctp-depleted RGC axons. Box plot whiskers indicate 5th-95th percentile. Right: Mean±s.e.m.; *P<0.0117, Mann–Whitney test. (I) Analysis of fast mitochondrial transport. Mean±s.e.m.; anterograde direction, *P*=0.9468; retrograde direction, *P*=0.7308; Mann–Whitney test. (J) Average duration of mitochondrial pauses in control and Tctp-depleted RGC axons. Box plot whiskers indicate 5-95 percentile and ‘+’ the mean; *P*=0.902, Mann–Whitney test. Permanently stationary mitochondria were excluded from this analysis. (K) Average number and frequency distributions of mitochondrial pauses. Mean±s.e.m.; *P*=0.317, Mann–Whitney test. Scale bars: 50 μm in E; 5 μm in F. n.s., not significant.

Because new mitochondria are generated in the neuronal soma, being transported from there to the cell periphery (Sheng and Cai, 2012), we reasoned that the decrease in axonal mitochondrial density observed in Tctp morphants could arise through impaired global mitochondrial biogenesis. We documented comparable mitochondrial DNA copy numbers (i.e. the ratio of mitochondrial to nuclear DNA) in Tctp-depleted retinas, as evaluated by quantitative PCR (Fig. 7A). Western blot analysis of Tctp-depleted tissues showed, in addition, unaltered expression levels of peroxisome proliferator-activated receptor gamma, coactivator 1 alpha (Pgc1α; Ppargc1a – Xenbase), a master inducer of mitochondrial biogenesis and regulator of mitochondrial density in neurons (Wareski et al., 2009) (Fig. 7B; Fig. S4C-E). In agreement with these findings, the expression levels of the nuclear-encoded mitochondrial genes examined [isocitrate dehydrogenase 3 (NAD^+^) alpha (*idh3a*); cytochrome c oxidase subunit Va (*cox5a*); cytochrome c, somatic (*cycs*); ras homolog family member T1 (*rhot1*) – Xenbase; also known as mitochondrial Rho GTPase 1 (*miro1*)] were unchanged relative to control retinas (Fig. 7C,D). Additionally, we detected similar *cox5a* mRNA expression levels in the GCL and in the IPL (made up of RGC dendrites and processes of other retinal neurons) in both backgrounds (Fig. 7E). Taken together, these data strongly indicate that mitochondrial biogenesis and mass are unaffected in Tctp morphants.

Having excluded impaired mitochondrial biogenesis, and because mitochondrial transport depends on mitochondrial function (Miller and Sheetz, 2004; Rintoul et al., 2003; Zala et al., 2013), we next investigated whether Tctp deficiency affects mitochondrial dynamics in axons. Analysis of 5-min time-lapse movies of labelled mitochondria showed a higher proportion of mitochondria moving in the retrograde direction (8.7% versus 1.9% in controls, *P=0.0358) and fewer mitochondria moving anterogradely (21.7% versus 35.2% in controls, *P=0.0353). In addition, the mean net displacement of mitochondria, including stationary, anterogradely trafficked and retrogradely trafficked organelles, was smaller in Tctp-depleted axons, although the bias was still in the anterograde direction (on average, each mitochondrion moved distally +4.4 μm compared with +8.9 μm in controls; Fig. 7F-H). However, the velocity of mitochondrial transport in the anterograde and retrograde directions, as well as the frequency and duration of mitochondrial pauses, were not significantly different between the groups (Fig. 7I-K; Fig. S4F), suggesting that the mitochondrial transport machinery is not compromised in Tctp morphants.

### Tctp acts via the survival machinery to promote axon development

Several studies indicate that Tctp interacts with members of the B-cell lymphoma 2 (Bcl2) family of proteins, which function as key mediators of mitochondrial integrity and apoptosis (Czabotar et al., 2014). Interestingly, the Bcl2 family is implicated in many instances of photoreceptor disease (Chen et al., 1996; Nir et al., 2000; Yang et al., 2004), and embryonic sensory neurons depleted of Bcl2, the prototypic member of this family, have reduced axon growth rates (Hilton et al., 1997), a phenotype we observe in Tctp morphants. Particularly well corroborated is the association of Tctp with Mcl1 (Liu et al., 2005; Yang et al., 2005; Zhang et al., 2002), a pro-survival Bcl2-related factor linked to neuroprotection responses in the CNS (Mori et al., 2004), prompting us to explore a potential interaction between these proteins in neurons.

**Fig. 8.**
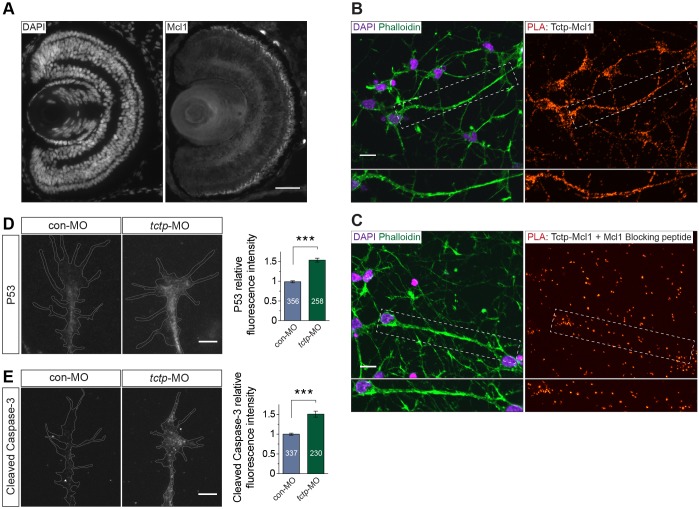
**Axonal Tctp interacts with pro-survival Mcl1.** (A) Coronal section of stage 43 retina probed with an anti-Mcl1 antibody and counterstained with DAPI. (B,C) PLA signal for Tctp and Mcl1 in cultured rat cortical neurons (E18.5+3 DIV) counterstained with DAPI and phalloidin. The boxed areas are enlarged beneath. In C, anti-Mcl1 serum and blocking peptide were co-incubated before proceeding with the assay. (D) Representative control and Tctp morphant RGC growth cones stained for P53. Mean±s.e.m.; *n*, number of growth cones analysed; ***P=0.0002, unpaired *t*-test. (E) Representative control and Tctp morphant RGC growth cones stained with an antibody that specifically recognises the cleaved (activated) form of Caspase-3. Mean±s.e.m.; *n*, number of growth cones analysed; ***P=0.0002, unpaired *t*-test. Scale bars: 50 μm in A; 10 μm in B,C; 5 μm in D,E.

First, we investigated whether Mcl1 is expressed *in vivo* by RGCs using an antibody raised against the *X. laevis* protein (Tsuchiya and Yamashita, 2011). Similar to Tctp, Mcl1 is expressed in the IPL, the OPL and the inner segment of photoreceptors. Mcl1 is also present in the GCL, the OFL and the ONH, indicating that Mcl1 localises to RGCs and their axons *in vivo* (Fig. 8A; Fig. S5A,B). In line with these data, Mcl1 was detected in the axonal and dendritic compartments of rat cortical neurons, confirming that, like Tctp, Mcl1 is present in neurites (Fig. S5C,D). We next tested whether Tctp physically interacts in axons with Mcl1 using a proximity ligation assay (PLA) (Söderberg et al., 2006; Yoon et al., 2012). We used rat cortical neurons in these studies owing to the availability of specific primary antibodies raised in different hosts, a central requirement of this methodology. Positive Tctp-Mcl1 PLA spots were abundantly detected in the cell body, but also along the neurites of cortical neurons [embryonic day (E) 18.5+3 DIV] (Fig. 8B,C; Fig. S6A), indicative of a close association between Tctp and Mcl1 (maximum working distance of the assay is in the range of 30-40 nm). We obtained an even more profuse signal in cultures aged *in vitro* for 14 days, suggesting that Tctp-Mcl1 interactions are not transient phenomena (Fig. S6B). Of particular note, ∼5-10% of Tctp-Mcl1-positive puncta colocalised with mitochondria in neurites (Fig. S6C). Collectively, these data validate previous biochemical reports and add a hitherto unexplored subcellular dimension to them, revealing that Tctp interacts with Mcl1 in the cell body and processes of neuronal cells.

Mechanistically, pro-survival members of the Bcl2 family (e.g. Mcl1) operate by sequestering pro-apoptotic proteins (e.g. Bax), thus preventing the release of cytochrome c from the mitochondrial intermembrane space and subsequent activation of caspases (Pease and Segal, 2014). Tctp is reported to stabilise and enhance Mcl1 biological activity (Liu et al., 2005) and to promote the degradation of P53 (tumor protein p53, Tp53 – Xenbase) (Amson et al., 2012; Rho et al., 2011), which itself neutralises the pro-survival actions of Bcl2 and Mcl1 at the mitochondria (Leu et al., 2004; Vaseva and Moll, 2009). We found using quantitative immunofluorescence that P53 expression was significantly upregulated in Tctp-depleted growth cones (Fig. 8D). In addition, we measured a 50% increase in active Caspase-3 mean signal relative to controls (Fig. 8E), consistent with a detrimental balance between pro- and anti-apoptotic signalling in Tctp morphants.

**Fig. 9.**
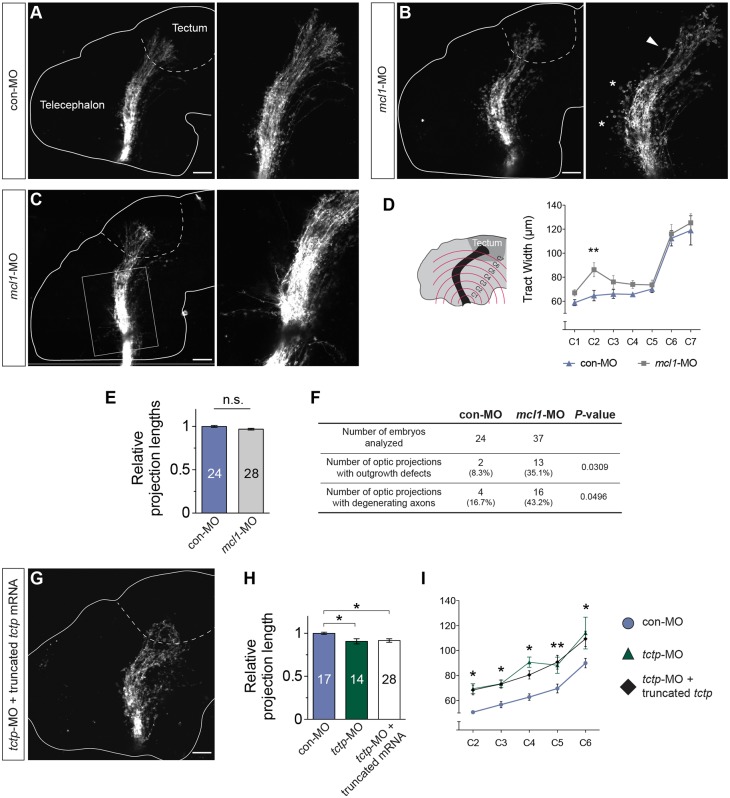
**Tctp regulates axon development via its anti-apoptotic effects.** (A-C) Lateral view of DiI-filled retinotectal projections in con-MO-injected or *mcl1*-MO-injected stage 40 embryos. Dashed lines approximate the boundary of the optic tectum; arrowhead denotes a region of the tract with outgrowth defects; asterisks mark beaded axons, suggestive of degenerating axons; boxed region in C shows axon misprojections into the telencephalon and diencephalon. Panels to the right show enlarged images. The boxed area in C is centred in the ventral optic tract. (D) Mean (±s.e.m.) optic tract width in con-MO-injected and *mcl1*-MO-injected embryos. C2, **P<0.01, two-way ANOVA. C2-7 denote imaginary, evenly spaced hemi-circumferences centred on the optic chiasm. (E) Relative projection lengths in control and Mcl1 morphant backgrounds. Mean±s.e.m.; *n*, number of brains analysed; n.s., not significant; Mann–Whitney test. (F) Summary of phenotypic changes in Mcl1 morphant projections (statistical significance determined using Fisher's exact test). (G) Co-delivery of *tctp*-MO and *tctp*_40-172_ mRNA, which encodes a truncated Tctp protein devoid of anti-apoptotic activity, fails to rescue the effects of Tctp depletion on the development of the retinotectal projection. (H) Relative projection lengths in embryos injected with con-MO, *tctp*-MO or *tctp*-MO+truncated *tctp*_40-172_ mRNA. Mean±s.e.m.; *n*, number of brains analysed; *P=0.008, Kruskal–Wallis test. (I) Mean (±s.e.m.) optic tract widths. con-MO versus *tctp*-MO+truncated *tctp*_40-172_ mRNA, *P<0.05 (C2), *P<0.05 (C3), *P<0.05 (C4), **P<0.01 (C5), *P<0.05 (C6), two-way ANOVA with Bonferroni correction. Scale bars: 50 μm.

Finally, we investigated whether Tctp acts via Mcl1 and the survival machinery to promote axon development. The retinotectal projection in Mcl1 morphants was found to be significantly wider along the ventral optic tract relative to controls, and axons often extended erroneously into the telencephalon (Fig. 9A-D; Fig. S6D). Furthermore, we detected outgrowth defects in subsets of axons coursing through the dorsal optic tract, although the absolute length of the projection was comparable to controls (Fig. 9C,E,F). We also noted a high frequency of degenerating axon profiles, distinguished by their beaded morphology, similar to that observed in Tctp morphants (Fig. 9B,F; Fig. S7A-D). Overall, these results suggest that Tctp and Mcl1 are functionally related, despite the qualitatively milder phenotypes detected in Mcl1 morphants. This might be due to compensation by other Bcl2-related proteins, as our data indicate that Tctp also interacts with Bcl-X_L_ in neurons (Fig. S8A,B).

To test directly whether Tctp pro-survival interactions are required for retinal axon development, we designed a mutated *tctp* rescue transgene encoding an N-terminally truncated Tctp protein lacking anti-apoptotic properties (Tctp_40-172aa_). Tctp_40-172aa_ retains Tctp signature motifs and the interaction domains of several known Tctp-interacting proteins (Yang et al., 2005), but not those necessary for the association with Mcl1 and Bcl-X_L_ (Yang et al., 2005; Zhang et al., 2002). Delivery of *tctp*_40-172_ mRNA together with *tctp*-MO by blastomere microinjection failed to mitigate the effects of Tctp depletion on the development of the retinotectal projection in terms of both tract length and tract width (Fig. 9G-I; Fig. S8C). Collectively, the findings are consistent with Tctp pro-survival actions being necessary for the normal development of the retinotectal projection.

## DISCUSSION

Uncontrolled growth and heightened survival are hallmarks of malignancy, allowing cancer cells to out-compete their neighbours and eventually dominate tissues. Tctp has previously been associated with cell growth, including during bone development and cancer pathogenesis (Amson et al., 2012; Brioudes et al., 2010; Kaarbo et al., 2013; Miao et al., 2013; Zhang et al., 2008), and is suggested to function as a pro-survival factor through its interplay with the Bcl2 protein family (Liu et al., 2005; Susini et al., 2008; Yang et al., 2005; Zhang et al., 2002). Thus, Tctp upregulation, as described in a variety of malignant tumours (Amson et al., 2012; Lo et al., 2012; Miao et al., 2013), is likely to reflect the growth and survival advantages that Tctp confers to the cell. In line with the many parallels that can be drawn between the normal processes of migratory growth cones during axon development and the disease mechanisms of cancer cell invasion, we document here that Tctp regulates the development of the retinotectal projection by impacting on axon growth and guidance, and link Tctp to the survival machinery of the axon.

Tctp deficiency leads to multiple mitochondria-related abnormalities in axons, including a substantially diminished mitochondrial membrane potential and decreased mitochondrial density. This evidence indicates that axonal Tctp contributes to the maintenance of mitochondrial function in this subcellular domain. Unlike vesicular fast axonal transport, which is reliant on the glycolytic pathway for its energetic needs (Zala et al., 2013), the trafficking of mitochondria is dependent on ATP generated by oxidative phosphorylation (Rintoul et al., 2003; Zala et al., 2013). Thus, considering the disruption of the mitochondrial membrane potential observed in Tctp-depleted axons, a parameter that directly influences mitochondrial ATP production, the defective accumulation of mitochondria at the neuronal periphery is perhaps a predictable outcome of compromised mitochondrial operation. Significantly, the general reduction in axonal mitochondrial density detected in these axons is not accompanied by alterations in mitochondrial biogenesis or mass, arguing that this deficit does not result from an inability of the neuron to generate mitochondria. Although we did not address the potential involvement of axonal mitophagy (Ashrafi et al., 2014), our analysis also indicates that more mitochondria are trafficked retrogradely in Tctp-depleted axons. Consistent with these findings, previously reported evidence indicates that dysfunctional mitochondria are selectively returned to the cell body for repair and/or degradation (Miller and Sheetz, 2004; Sheng and Cai, 2012). Hence, the insult to mitochondria in axons depleted of Tctp might, in effect, lead to a secondary perturbation on mitochondrial dynamics and an overall more prominent decline in axonal mitochondrial distribution.

How does Tctp promote mitochondrial function? Pro-survival members of the Bcl2 protein family, such as Mcl1, work primarily by sequestering and neutralising Bcl2-related pro-apoptotic factors (e.g. Bax), which, if left uncontrolled, negatively affect the integrity of mitochondria (Shamas-Din et al., 2013). A fitting analogy would be a molecular tug-of-war between pro- and anti-apoptotic Bcl2-related factors controlling mitochondrial homeostasis. According to the model put forward by Susini and colleagues, Tctp pro-survival activity results from its blocking Bax dimerisation, a key mitochondrial outer membrane permeabilisation (MOMP)-inducing event, by binding and reconfiguring Mcl1 and Bcl-X_L_ in such a way that their inhibitory actions on Bax are promoted (Susini et al., 2008). Akin to the pivotal role of mitochondria in neutrophil chemotaxis (Bao et al., 2015), we speculate that compromised pro-survival signalling in axons deficient in Tctp translates into mitochondrial dysfunction and a secondary decline in axonal mitochondrial density, ultimately resulting in an energy and Ca^2+^-buffering state insufficient to sustain the normal processes of a growing axon. These effects would be particularly acute in the growth cone, an ATP-intensive distal outpost where mitochondria accumulate (Lathrop and Steketee, 2013), impairing its ability to adequately respond to guidance and growth-promoting cues in the embryonic environment.

In summary, the findings presented here suggest that Tctp functions as a checkpoint for the normal development of the retinotectal projection via its regulation of pro-survival signalling and axonal mitochondrial homeostasis. Although the precise role(s) of mitochondria in axon growth and navigation are still unresolved, it will also be interesting in the future to investigate the possibility that Tctp regulates mechanisms extrinsic to mitochondria. Indeed, considering the involvement of local caspase action in axon guidance and branching behaviours (Campbell and Holt, 2003; Campbell and Okamoto, 2013; Ohsawa et al., 2010), future work should address the contribution of dysfunctional caspase activation towards the axon development defects observed in Tctp morphant embryos.

## MATERIALS AND METHODS

### *Xenopus laevis* embryos

*Xenopus laevis* embryos of either sex were obtained by *in vitro* fertilisation, raised in 0.1× Modified Barth's Saline [0.88 mM NaCl, 0.01 mM KCl, 0.024 mM NaHCO_3_, 0.1 mM HEPES, 8.2 μM MgSO_4_, 3.3 μM Ca(NO_3_)_2_, 4.1 μM CaCl_2_] at 14-18°C, and staged following Nieuwkoop and Faber (Nieuwkoop and Faber, 1994). All animal experiments were approved by the University of Cambridge Ethical Review Committee.

### Retinal cultures

Unless otherwise noted, eye primordia were dissected from anaesthetised stage 32 larvae, and plated on culture dishes coated with poly-L-lysine (10 μg/ml, Sigma) and laminin (10 μg/ml, Sigma). Cultures were incubated at 20°C in 60% L15 minimal medium (Life Technologies) for 24 h before further manipulation.

### Immunostaining of retinal sections

Stage 43 (unless otherwise specified) transverse 12-μm cryosections were processed using standard immunohistochemistry procedures (blocking solution: 10% heat-inactivated goat serum, 1% bovine serum albumin, 0.5% Triton X-100 in 1× PBS). Antigen retrieval with steaming 0.01 M sodium citrate (0.05% Tween 20, pH 6.0) was carried out before staining for Tctp. For further details, including antibodies, see supplementary Materials and Methods.

### *In situ* hybridisation and fluorescence *in situ* hybridisation

ISH on retinal sections was performed as described previously (Xue and Harris, 2012) using digoxigenin (DIG)-labelled riboprobes generated from IMAGE clones. Four non-overlapping DNA oligonucleotides complementary to the *tctp* coding sequence were DIG-labelled and hybridisation procedure on retinal growth cones carried out as previously described (Zivraj et al., 2010). For further details, including probe sequences, see supplementary Materials and Methods.

### Eye RNA extraction

Retinas from stage 37/38 embryos were dissected in 1× Modified Barth's Saline containing ethyl 3-aminobenzoate methanesulfonate (0.04% wt/vol; Sigma) and put on ice. Total RNA extraction was performed using a column-based purification method following the instructions provided by the manufacturer (RNeasy Mini Kit, Qiagen). Tissue homogenisation was achieved by vortexing.

### Laser-capture microdissection and RNA extraction

Per experiment, ∼140 stage 33/34 eye explants were plated on polyethylene terephthalate slides, pre-coated with poly-L-lysine (10 μg/ml) and laminin (10 μg/ml), and cultured for 32-36 h. Retinal cultures were fixed (4% paraformaldehyde, 4% sucrose in 1× PBS) for 10 min, dehydrated through an ethanol series, and air-dried before the microdissection procedure, as described previously (Zivraj et al., 2010). RNA was extracted using the RNAqueous-Micro Kit (Life Technologies) according to the manufacturer's instructions. Extract purity was determined by RT-PCR. For further details, see supplementary Materials and Methods.

### 5′ and 3′ rapid amplification of cDNA ends (RACE PCR)

RNA extracts were processed with SMARTer RACE cDNA Amplification Kit (Clontech) according to the manufacturer's instructions. RACE PCR was performed using Advantage HF 2 PCR Kit (Clontech). Following gel extraction, PCR products were TA-cloned and sequenced in both orientations.

### Morpholino oligonucleotides

Antisense *tctp*-MO (translation-blocking), *mcl1*-MO (splice-blocking) and control-MO were supplied by GeneTools: 5′-ATCATGTTGGCGGCCTAAGTGTTGT-3′, 5′-AGTAGAGTAAGCCATGCTCACCCGT-3′ and 5′-CCTCTTACCTCAGTTACAATTTATA-3′, respectively. For further details, see supplementary Materials and Methods.

### Blastomere microinjection

Dorsal blastomere injections were performed at the four-cell stage as described previously (Leung and Holt, 2008). *tctp*-MO and *mcl1*-MO were injected at 12 ng/blastomere and 6 ng/blastomere, respectively.

### Retina-targeted electroporation

Plasmid DNA electroporation was carried out on stage 28-30 embryos as described previously (Falk et al., 2007) using eight consecutive 18 V pulses of 50 ms duration, delivered at 1-s intervals. gap-RFP and mt-GFP were delivered at 1 mg/μl.

### DiI labelling of retinal axons

Embryos were fixed overnight at 4°C in 4% paraformaldehyde in PBS and RGC axons labelled by intraocular injection of the fluorescent carbocyanine DiI. The contralateral (with respect to the dye-injected eye) brain hemisphere was later dissected, mounted in 1× PBS and visualised using confocal microscopy. Tract length was normalised to the distance between the optic chiasm and the posterior boundary of the tectum. For further details, see supplementary Materials and Methods.

### *In vivo* imaging of axon pathfinding

Live imaging of pathfinding retinal axons was performed as described previously (Leung et al., 2013). Specimens were mounted in an imaging chamber constructed on oxygenated Permanox slides (Nunc). Images were acquired every 15 min for 2 h. Axons were scored as ‘stalled’ if their outgrowth was ≤10 μm over the 2-h period of analysis. For further details, see supplementary Materials and Methods.

### Analysis of photoreceptor degeneration

Transverse stage 43 retinal sections probed for opsin or rhodopsin and counterstained with DAPI were used, respectively, in cone and rod photoreceptor phenotypic analyses. The average distance between the fluorescence signals in the outer nuclear layer (i.e. DAPI-stained photoreceptor cell bodies) and the photoreceptor outer segments was taken as an approximation of photoreceptor inner segment length. The width of the gaps left open by collapsed outer segments was normalised to the perimeter of the outer nuclear layer to estimate the percentage of photoreceptors with degenerative phenotypes. Cone and rod analyses were conducted independently.

### TUNEL assay

TUNEL labelling of stage 43 transverse 12-μm cryosections was performed following the instructions provided by the manufacturer (*In situ* Cell Death Detection Kit – TMR red, Roche). Data measurements reflect, per section, the ratio between TUNEL-positive nuclei and the total number of DAPI-stained nuclei in the ganglion cell layer. For further details, see supplementary Materials and Methods.

### ATP bioluminescence assay

ATP content was measured using the ATP Bioluminescence Kit CLS II (Roche). Immediately after dissection, single retinas were incubated in 50 μl of 1% perchloric acid for precisely 10 min at room temperature before the reaction was stopped in 450 μl of boiling Tris buffer (100 mM Tris, 4 mM EDTA, pH 7.75), incubated for 2 min at 100°C, and centrifuged at 1000 ***g*** for 1 min. For further details, see supplementary Materials and Methods.

### Mitochondrial membrane potential assessment

Retinal cultures were incubated with 20 nM tetramethylrhodamine, methyl ester (TMRM) at 20°C for 20 min and washed with culture medium before imaging. ΔΨ_p_-corrected ΔΨ_m_ measurements were derived from the ratio of fluorescence intensities between mitochondria (*F_m_*) and mitochondria-poor regions (*F_c_*) (Marks et al., 2005). For further details, see supplementary Materials and Methods.

### Visualising mitochondrial dynamics

Retinal explants were incubated with 25 nM MitoTracker Red (Life Technologies) at 20°C for 20 min and washed with culture medium before imaging. Time-lapse recordings were run for 5 min applying 5-s intervals between time points. A mobile mitochondrion was only considered as such if its dislocation was ≥5 μm (Sheng and Cai, 2012). The subset of mitochondria undergoing fast transport were defined as those moving at average velocities of ≥0.3 μm/s. For further details, see supplementary Materials and Methods.

### Real-time PCR

Per condition, seven to nine independent samples were collected on different days each consisting of two retinas dissected from the same embryo (stage 37/38). When quantifying nuclear-encoded mitochondria-related targets, reference gene (*ywhaz*, *rps13*, *hprt1*, *tbp1*) normalisation was performed using geNorm; the optimal normalisation factor was calculated as the geometric mean of reference targets *ywhaz* and *tbp1* and data analysis performed within qbase+ (Biogazelle). Mitochondrial DNA content determination used *gcg* and *b2m* (genomic targets), and *mt-tl1* and *atp6* (mitochondria-encoded gene targets). Mitochondrial DNA content and RNA isoform data were analysed using the ΔΔCq method. For further details, see supplementary Materials and Methods and Table S1.

### Primary rat cortex neuronal culture and immunocytochemistry

Foetal neurons derived from cortices of F344 rat E18.5 embryos were obtained from Cyagen Biosciences as cryopreserved primary cells, and plated on culture dishes pre-coated with poly-L-lysine (15 μg/ml, Sigma) and laminin (15 μg/ml, Sigma). Neuronal cultures were grown at 37°C in a 5% CO_2_ humidified incubator in OriCell Neuron Growth Medium (Cyagen Biosciences) supplemented with L-alanyl-L-glutamine (Life Technologies) and B-27 (Life Technologies) for at least 72 h before further manipulation. For immunocytochemistry, cells were fixed, washed and permeabilised then standard protocols followed. For immunocytochemistry, cells were fixed in pre-warmed 4% paraformaldehyde, washed with 10 mM glycine in 1× PBS, and permeabilised with 0.03% Triton X-100 in 1× PBS. Images were acquired using a laser scanning confocal microscope. For further details, including antibodies, see supplementary Materials and Methods.

### *In situ* proximity ligation assay

*In situ* proximity ligation assays were performed on rat cortical neurons or HCT116 cells (certified by ATCC) according to manufacturer's recommendations (Duolink; Olink Biosciences). The following primary antibody pairs were used: mouse monoclonal anti-Tctp (1:400; Santa Cruz Biotechnology, sc-133131) and rabbit polyclonal anti-Mcl1 (1:100; Santa Cruz Biotechnology, sc-819); mouse anti-Tctp (1:400; Santa Cruz Biotechnology, sc-133131) and rabbit anti-Bcl-X_L_ (1:100; Santa Cruz Biotechnology, sc-7195). Additionally, a blocking Mcl1 peptide (provided by Santa Cruz Biotechnology), used at a fivefold excess relative to the anti-Mcl1 antibody, was included in preliminary experiments to evaluate the specificity of the technique. For further details, see supplementary Materials and Methods.

### Quantitative immunofluorescence

Quantitative immunofluorescence was performed as described previously (Leung et al., 2013). Background-corrected fluorescence intensities (mean pixel intensity per unit area) were measured in non-collapsed growth cones. For further details, see supplementary Materials and Methods.

### Western blot

Stage 35/36 (unless otherwise specified) eye and/or brain lysates were prepared in ice-cold RIPA buffer and resolved by SDS-PAGE. Both ‘semi-dry’ and ‘wet’ electroblotting methods were applied, depending on the size of the target protein to be analysed. After electroblotting, nitrocellulose membranes were probed using the following primary antibodies: anti-Tctp (1:5000; gift from J. Kubiak, Université de Rennes, France); anti-Pgc1α (1:500; Aviva Systems Biology, ARP39015_P050) and anti-Cytochrome-c (1:1000; Invitrogen, 338500). HRP-conjugated secondary antibodies (Abcam) were used in combination with a chemiluminescence-based detection system (Amersham ECL, GE Healthcare). To evaluate the efficiency of Tctp knockdown in HCT116 cell lines, a commercially available anti-Tctp antibody was used (1:500; Santa Cruz Biotechnology, sc-133131).

### Statistical analysis

Each experiment was repeated at least three times unless otherwise indicated. Details of statistical analysis are included in figure legends or main text. Data were analysed with Prism (GraphPad), except real-time PCR data (qbase+). For all tests, a significance threshold of α=0.05 was used.

### Note added in proof

New research indicates that Tctp has a BH3-like domain which potentiates, rather than inhibits, the anti-apoptotic function of Bcl-XL (Thebault et al., 2016).

## References

[DEV131060C1] Agathocleous M., Love N. K., Randlett O., Harris J. J., Liu J., Murray A. J. and Harris W. A. (2012). Metabolic differentiation in the embryonic retina. *Nat. Cell Biol.* 14, 859-864. 10.1038/ncb2531 22750943 PMC3442239

[DEV131060C2] Alexander C., Votruba M., Pesch U. E. A., Thiselton D. L., Mayer S., Moore A., Rodriguez M., Kellner U., Leo-Kottler B., Auburger G. et al. (2000). OPA1, encoding a dynamin-related GTPase, is mutated in autosomal dominant optic atrophy linked to chromosome 3q28. *Nat. Genet.* 26, 211-215. 10.1038/79944 11017080

[DEV131060C3] Amson R., Pece S., Lespagnol A., Vyas R., Mazzarol G., Tosoni D., Colaluca I., Viale G., Rodrigues-Ferreira S., Wynendaele J. et al. (2012). Reciprocal repression between P53 and TCTP. *Nat. Med.* 18, 91-99. 10.1038/nm.2546 22157679

[DEV131060C4] Andreassi C., Zimmermann C., Mitter R., Fusco S., De Vita S., Saiardi A. and Riccio A. (2010). An NGF-responsive element targets myo-inositol monophosphatase-1 mRNA to sympathetic neuron axons. *Nat. Neurosci.* 13, 291-301. 10.1038/nn.2486 20118926

[DEV131060C5] Ashrafi G., Schlehe J. S., LaVoie M. J. and Schwarz T. L. (2014). Mitophagy of damaged mitochondria occurs locally in distal neuronal axons and requires PINK1 and Parkin. *J. Cell Biol.* 206, 655-670. 10.1083/jcb.201401070 25154397 PMC4151150

[DEV131060C6] Bao Y., Ledderose C., Graf A. F., Brix B., Birsak T., Lee A., Zhang J. and Junger W. G. (2015). mTOR and differential activation of mitochondria orchestrate neutrophil chemotaxis. *J. Cell Biol.* 210, 1153-1164. 10.1083/jcb.201503066 26416965 PMC4586745

[DEV131060C7] Bassell G. J., Zhang H., Byrd A. L., Femino A. M., Singer R. H., Taneja K. L., Lifshitz L. M., Herman I. M. and Kosik K. S. (1998). Sorting of beta-actin mRNA and protein to neurites and growth cones in culture. *J. Neurosci.* 18, 251-265. 9412505 10.1523/JNEUROSCI.18-01-00251.1998 PMC6793411

[DEV131060C8] Bazile F., Pascal A., Arnal I., Le Clainche C., Chesnel F. and Kubiak J. Z. (2009). Complex relationship between TCTP, microtubules and actin microfilaments regulates cell shape in normal and cancer cells. *Carcinogenesis* 30, 555-565. 10.1093/carcin/bgp022 19168579 PMC2831045

[DEV131060C9] Benndorf R., Nürnberg P. and Bielka H. (1988). Growth phase-dependent proteins of the Ehrlich ascites tumor analyzed by one- and two-dimensional electrophoresis. *Exp. Cell Res.* 174, 130-138. 10.1016/0014-4827(88)90148-6 3335219

[DEV131060C10] Biankin A. V., Waddell N., Kassahn K. S., Gingras M.-C., Muthuswamy L. B., Johns A. L., Miller D. K., Wilson P. J., Patch A.-M., Wu J. et al. (2012). Pancreatic cancer genomes reveal aberrations in axon guidance pathway genes. *Nature* 491, 399-405. 10.1038/nature11547 23103869 PMC3530898

[DEV131060C11] Bohm H., Benndorf R., Gaestel M., Gross B., Nurnberg P., Kraft R., Otto A. and Bielka H. (1989). The growth-related protein P23 of the Ehrlich ascites tumor: translational control, cloning and primary structure. *Biochem. Int.* 19, 277-286. 2479380

[DEV131060C12] Brioudes F., Thierry A.-M., Chambrier P., Mollereau B. and Bendahmane M. (2010). Translationally controlled tumor protein is a conserved mitotic growth integrator in animals and plants. *Proc. Natl. Acad. Sci. USA* 107, 16384-16389. 10.1073/pnas.1007926107 20736351 PMC2941279

[DEV131060C13] Campbell D. S. and Holt C. E. (2003). Apoptotic pathway and MAPKs differentially regulate chemotropic responses of retinal growth cones. *Neuron* 37, 939-952. 10.1016/S0896-6273(03)00158-2 12670423

[DEV131060C14] Campbell D. S. and Okamoto H. (2013). Local caspase activation interacts with Slit-Robo signaling to restrict axonal arborization. *J. Cell Biol.* 203, 657-672. 10.1083/jcb.201303072 24385488 PMC3840933

[DEV131060C15] Chen J., Flannery J. G., LaVail M. M., Steinberg R. H., Xu J. and Simon M. I. (1996). bcl-2 overexpression reduces apoptotic photoreceptor cell death in three different retinal degenerations. *Proc. Natl. Acad. Sci. USA* 93, 7042-7047. 10.1073/pnas.93.14.7042 8692941 PMC38932

[DEV131060C16] Chen S. H., Wu P.-S., Chou C.-H., Yan Y.-T., Liu H., Weng S.-Y. and Yang-Yen H.-F. (2007). A knockout mouse approach reveals that TCTP functions as an essential factor for cell proliferation and survival in a tissue- or cell type-specific manner. *Mol. Biol. Cell* 18, 2525-2532. 10.1091/mbc.E07-02-0188 17475776 PMC1924818

[DEV131060C17] Czabotar P. E., Lessene G., Strasser A. and Adams J. M. (2014). Control of apoptosis by the BCL-2 protein family: implications for physiology and therapy. *Nat. Rev. Mol. Cell Biol.* 15, 49-63. 10.1038/nrm3722 24355989

[DEV131060C18] Delettre C., Lenaers G., Griffoin J.-M., Gigarel N., Lorenzo C., Belenguer P., Pelloquin L., Grosgeorge J., Turc-Carel C., Perret E. et al. (2000). Nuclear gene OPA1, encoding a mitochondrial dynamin-related protein, is mutated in dominant optic atrophy. *Nat. Genet.* 26, 207-210. 10.1038/79936 11017079

[DEV131060C19] Di Giammartino D. C., Nishida K. and Manley J. L. (2011). Mechanisms and consequences of alternative polyadenylation. *Mol. Cell* 43, 853-866. 10.1016/j.molcel.2011.08.017 21925375 PMC3194005

[DEV131060C20] Falk J., Drinjakovic J., Leung K. M., Dwivedy A., Regan A. G., Piper M. and Holt C. E. (2007). Electroporation of cDNA/Morpholinos to targeted areas of embryonic CNS in Xenopus. *BMC Dec. Biol.* 7, 107 10.1186/1471-213X-7-107 PMC2147031 17900342

[DEV131060C21] Fountoulakis M., Berndt P., Langen H. and Suter L. (2002). The rat liver mitochondrial proteins. *Electrophoresis* 23, 311-328. 10.1002/1522-2683(200202)23:2<311::AID-ELPS311>3.0.CO;2-0 11840540

[DEV131060C22] Gumy L. F., Yeo G. S. H., Tung Y.-C. L., Zivraj K. H., Willis D., Coppola G., Lam B. Y. H., Twiss J. L., Holt C. E. and Fawcett J. W. (2011). Transcriptome analysis of embryonic and adult sensory axons reveals changes in mRNA repertoire localization. *RNA* 17, 85-98. 10.1261/rna.2386111 21098654 PMC3004069

[DEV131060C23] Hilton M., Middleton G. and Davies A. M. (1997). Bcl-2 influences axonal growth rate in embryonic sensory neurons. *Curr. Biol.* 7, 798-800. 10.1016/S0960-9822(06)00339-3 9368763

[DEV131060C24] Holt C. E. and Harris W. A. (1983). Order in the initial retinotectal map in Xenopus: a new technique for labelling growing nerve fibres. *Nature* 301, 150-152. 10.1038/301150a0 6823290

[DEV131060C25] Hsu Y.-C., Chern J. J., Cai Y., Liu M. and Choi K.-W. (2007). Drosophila TCTP is essential for growth and proliferation through regulation of dRheb GTPase. *Nature* 445, 785-788. 10.1038/nature05528 17301792

[DEV131060C26] Kaarbø M., Storm M. L., Qu S., Waehre H., Risberg B., Danielsen H. E. and Saatcioglu F. (2013). TCTP is an androgen-regulated gene implicated in prostate cancer. *PLoS ONE* 8, e69398 10.1371/journal.pone.0069398 23894469 PMC3718683

[DEV131060C27] Kamath R. S., Fraser A. G., Dong Y., Poulin G., Durbin R., Gotta M., Kanapin A., Le Bot N., Moreno S., Sohrmann M. et al. (2003). Systematic functional analysis of the Caenorhabditis elegans genome using RNAi. *Nature* 421, 231-237. 10.1038/nature01278 12529635

[DEV131060C28] Kim S. H., Cairns N., Fountoulakisc M. and Lubec G. (2001). Decreased brain histamine-releasing factor protein in patients with Down syndrome and Alzheimer's disease. *Neurosci. Lett.* 300, 41-44. 10.1016/S0304-3940(01)01545-2 11172935

[DEV131060C29] Kim M., Maeng J. and Lee K. (2013). Dimerization of TCTP and its clinical implications for allergy. *Biochimie* 95, 659-666. 10.1016/j.biochi.2012.10.007 23104268

[DEV131060C30] Lathrop K. L. and Steketee M. B. (2013). Mitochondrial dynamics in retinal ganglion cell axon regeneration and growth cone guidance. *J. Ocul. Biol.* 1, 9. 24616897 PMC3946936

[DEV131060C31] Leu J. I.-J., Dumont P., Hafey M., Murphy M. E. and George D. L. (2004). Mitochondrial p53 activates Bak and causes disruption of a Bak–Mcl1 complex. *Nat. Cell Biol.* 6, 443-450. 10.1038/ncb1123 15077116

[DEV131060C32] Leung K. M. and Holt C. E. (2008). Live visualization of protein synthesis in axonal growth cones by microinjection of photoconvertible Kaede into Xenopus embryos. *Nat. Protoc.* 3, 1318-1327. 10.1038/nprot.2008.113 18714300 PMC3687492

[DEV131060C33] Leung K.-M., van Horck F. P. G., Lin A. C., Allison R., Standart N. and Holt C. E. (2006). Asymmetrical beta-actin mRNA translation in growth cones mediates attractive turning to netrin-1. *Nat. Neurosci.* 9, 1247-1256. 10.1038/nn1775 16980963 PMC1997306

[DEV131060C34] Leung L. C., Urbancic V., Baudet M. L., Dwivedy A., Bayley T. G., Lee A. C., Harris W. A. and Holt C. E. (2013). Coupling of NF-protocadherin signaling to axon guidance by cue-induced translation. *Nat. Neurosci.* 16, 166-173. 10.1038/nn.3290 23292679 PMC3701881

[DEV131060C35] Liu H., Peng H.-W., Cheng Y.-S., Yuan H. S. and Yang-Yen H.-F. (2005). Stabilization and enhancement of the antiapoptotic activity of mcl-1 by TCTP. *Mol. Cell. Biol.* 25, 3117-3126. 10.1128/MCB.25.8.3117-3126.2005 15798198 PMC1069602

[DEV131060C36] Lo W.-Y., Wang H.-J., Chiu C.-W. and Chen S.-F. (2012). miR-27b-regulated TCTP as a novel plasma biomarker for oral cancer: from quantitative proteomics to post-transcriptional study. *J. Proteomics* 77, 154-166. 10.1016/j.jprot.2012.07.039 22902387

[DEV131060C37] MacDonald S. M., Rafnar T., Langdon J. and Lichtenstein L. M. (1995). Molecular identification of an IgE-dependent histamine-releasing factor. *Science* 269, 688-690. 10.1126/science.7542803 7542803

[DEV131060C38] Marks J. D., Boriboun C. and Wang J. (2005). Mitochondrial nitric oxide mediates decreased vulnerability of hippocampal neurons from immature animals to NMDA. *J. Neurosci.* 25, 6561-6575. 16014717 10.1523/JNEUROSCI.1450-05.2005 PMC6725441

[DEV131060C39] Martin K. C. and Ephrussi A. (2009). mRNA localization: gene expression in the spatial dimension. *Cell* 136, 719-730. 10.1016/j.cell.2009.01.044 19239891 PMC2819924

[DEV131060C40] Mehlen P., Delloye-Bourgeois C. and Chédotal A. (2011). Novel roles for Slits and netrins: axon guidance cues as anticancer targets? *Nat. Rev. Cancer* 11, 188-197. 10.1038/nrc3005 21326323

[DEV131060C41] Miao X., Chen Y.-B., Xu S.-L., Zhao T., Liu J.-Y., Li Y.-R., Wang J., Zhang J. and Guo G.-Z. (2013). TCTP overexpression is associated with the development and progression of glioma. *Tumour Biol.* 34, 3357-3361. 10.1007/s13277-013-0906-9 23749504

[DEV131060C42] Miller K. E. and Sheetz M. P. (2004). Axonal mitochondrial transport and potential are correlated. *J. Cell Sci.* 117, 2791-2804. 10.1242/jcs.01130 15150321

[DEV131060C43] Mori M., Burgess D. L., Gefrides L. A., Foreman P. J., Opferman J. T., Korsmeyer S. J., Cavalheiro E. A., Naffah-Mazzacoratti M. G. and Noebels J. L. (2004). Expression of apoptosis inhibitor protein Mcl1 linked to neuroprotection in CNS neurons. *Cell Death Differ.* 11, 1223-1233. 10.1038/sj.cdd.4401483 15286683

[DEV131060C44] Nieuwkoop P. D. and Faber J. (1994). Normal table of Xenopus laevis (Daudin) : a systematical and chronological survey of the development from the fertilized egg till the end of metamorphosis. New York: Garland Pub.

[DEV131060C45] Nir I., Kedzierski W., Chen J. and Travis G. H. (2000). Expression of Bcl-2 protects against photoreceptor degeneration in retinal degeneration slow (rds) mice. *J. Neurosci.* 20, 2150-2154. 10704489 10.1523/JNEUROSCI.20-06-02150.2000 PMC6772515

[DEV131060C46] Nunnari J. and Suomalainen A. (2012). Mitochondria: in sickness and in health. *Cell* 148, 1145-1159. 10.1016/j.cell.2012.02.035 22424226 PMC5381524

[DEV131060C47] Ohsawa S., Hamada S., Kuida K., Yoshida H., Igaki T. and Miura M. (2010). Maturation of the olfactory sensory neurons by Apaf-1/caspase-9-mediated caspase activity. *Proc. Natl. Acad. Sci. USA* 107, 13366-13371. 10.1073/pnas.0910488107 20624980 PMC2922127

[DEV131060C48] Ong C. K., Subimerb C., Pairojkul C., Wongkham S., Cutcutache I., Yu W., McPherson J. R., Allen G. E., Ng C. C., Wong B. H. et al. (2012). Exome sequencing of liver fluke–associated cholangiocarcinoma. *Nat. Genet.* 44, 690-693. 10.1038/ng.2273 22561520

[DEV131060C49] Pagano G. and Castello G. (2012). Oxidative stress and mitochondrial dysfunction in Down syndrome. *Adv. Exp. Med. Biol.* 724, 291-299. 10.1007/978-1-4614-0653-2_22 22411251

[DEV131060C50] Pasquale E. B. (2010). Eph receptors and ephrins in cancer: bidirectional signalling and beyond. *Nat. Rev. Cancer* 10, 165-180. 10.1038/nrc2806 20179713 PMC2921274

[DEV131060C51] Pease S. E. and Segal R. A. (2014). Preserve and protect: maintaining axons within functional circuits. *Trends Neurosci.* 37, 572-582. 10.1016/j.tins.2014.07.007 25167775 PMC4245037

[DEV131060C52] Perry R. B.-T., Doron-Mandel E., Iavnilovitch E., Rishal I., Dagan S. Y., Tsoory M., Coppola G., McDonald M. K., Gomes C., Geschwind D. H. et al. (2012). Subcellular knockout of importin beta1 perturbs axonal retrograde signaling. *Neuron* 75, 294-305. 10.1016/j.neuron.2012.05.033 22841314 PMC3408616

[DEV131060C53] Rezaul K., Wu L., Mayya V., Hwang S. I. and Han D. (2005). A systematic characterization of mitochondrial proteome from human T leukemia cells. *Mol. Cell. Proteomics* 4, 169-181. 10.1074/mcp.M400115-MCP200 15598749 PMC1487188

[DEV131060C54] Rho S. B., Lee J. H., Park M. S., Byun H.-J., Kang S., Seo S.-S., Kim J.-Y. and Park S.-Y. (2011). Anti-apoptotic protein TCTP controls the stability of the tumor suppressor p53. *FEBS Lett.* 585, 29-35. 10.1016/j.febslet.2010.11.014 21081126

[DEV131060C55] Rintoul G. L., Filiano A. J., Brocard J. B., Kress G. J. and Reynolds I. J. (2003). Glutamate decreases mitochondrial size and movement in primary forebrain neurons. *J. Neurosci.* 23, 7881-7888. 10.1523/JNEUROSCI.1450-05.2005 12944518 PMC6740596

[DEV131060C56] Shamas-Din A., Kale J., Leber B. and Andrews D. W. (2013). Mechanisms of action of Bcl-2 family proteins. *Cold Spring Harb. Perspect. Biol.* 5, a008714 10.1101/cshperspect.a008714 23545417 PMC3683897

[DEV131060C57] Sheng Z.-H. and Cai Q. (2012). Mitochondrial transport in neurons: impact on synaptic homeostasis and neurodegeneration. *Nat. Rev. Neurosci.* 13, 77-93. 10.1038/nrn3156 22218207 PMC4962561

[DEV131060C58] Söderberg O., Gullberg M., Jarvius M., Ridderstråle K., Leuchowius K.-J., Jarvius J., Wester K., Hydbring P., Bahram F., Larsson L.-G. et al. (2006). Direct observation of individual endogenous protein complexes in situ by proximity ligation. *Nat. Methods* 3, 995-1000. 10.1038/nmeth947 17072308

[DEV131060C59] Susini L., Besse S., Duflaut D., Lespagnol A., Beekman C., Fiucci G., Atkinson A. R., Busso D., Poussin P., Marine J.-C. et al. (2008). TCTP protects from apoptotic cell death by antagonizing bax function. *Cell Death Differ.* 15, 1211-1220. 10.1038/cdd.2008.18 18274553

[DEV131060C60] Tamagnone L. (2012). Emerging role of semaphorins as major regulatory signals and potential therapeutic targets in cancer. *Cancer Cell* 22, 145-152. 10.1016/j.ccr.2012.06.031 22897846

[DEV131060C61] Taylor A. M., Berchtold N. C., Perreau V. M., Tu C. H., Li Jeon N. and Cotman C. W. (2009). Axonal mRNA in uninjured and regenerating cortical mammalian axons. *J. Neurosci.* 29, 4697-4707. 10.1523/JNEUROSCI.6130-08.2009 19369540 PMC3632375

[DEV131060C62] Tessier-Lavigne M. and Goodman C. S. (1996). The molecular biology of axon guidance. *Science* 274, 1123-1133. 10.1126/science.274.5290.1123 8895455

[DEV131060C163] Thebault S., Agez M., Chi X., Stojko J., Cura V., Telerman S. B., Maillet L., Gautier F., Billas-Massobrio I., Birck C. et al. (2016). TCTP contains a BH3-like domain, which instead of inhibiting, activates Bcl-xL. *Sci. Rep.* 6, 19725 10.1038/srep19725 26813996 PMC4728560

[DEV131060C63] Thiele H., Berger M., Skalweit A. and Thiele B.-J. (2000). Expression of the gene and processed pseudogenes encoding the human and rabbit translationally controlled tumour protein (TCTP). *Eur. J. Biochem.* 267, 5473-5481. 10.1046/j.1432-1327.2000.01609.x 10951206

[DEV131060C64] Tsuchiya Y. and Yamashita S. (2011). Anti-apoptotic activity and proteasome-mediated degradation of Xenopus Mcl-1 protein in egg extracts. *J. Biol. Chem.* 286, 15806-15814. 10.1074/jbc.M110.175927 21454490 PMC3091190

[DEV131060C65] Tuynder M., Susini L., Prieur S., Besse S., Fiucci G., Amson R. and Telerman A. (2002). Biological models and genes of tumor reversion: cellular reprogramming through tpt1/TCTP and SIAH-1. *Proc. Natl. Acad. Sci. USA* 99, 14976-14981. 10.1073/pnas.222470799 12399545 PMC137530

[DEV131060C66] Vaseva A. V. and Moll U. M. (2009). The mitochondrial p53 pathway. *Biochim. Biophys. Acta* 1787, 414-420. 10.1016/j.bbabio.2008.10.005 19007744 PMC2819081

[DEV131060C67] Wallace D. C., Singh G., Lott M. T., Hodge J. A., Schurr T. G., Lezza A. M., Elsas L. J. II and Nikoskelainen E. K. (1988). Mitochondrial DNA mutation associated with Leber's hereditary optic neuropathy. *Science* 242, 1427-1430. 10.1126/science.3201231 3201231

[DEV131060C68] Wareski P., Vaarmann A., Choubey V., Safiulina D., Liiv J., Kuum M. and Kaasik A. (2009). PGC-1{alpha} and PGC-1{beta} regulate mitochondrial density in neurons. *J. Biol. Chem.* 284, 21379-21385. 10.1074/jbc.M109.018911 19542216 PMC2755862

[DEV131060C69] Wright A. F., Chakarova C. F., Abd El-Aziz M. M. and Bhattacharya S. S. (2010). Photoreceptor degeneration: genetic and mechanistic dissection of a complex trait. *Nat. Rev. Genet.* 11, 273-284. 10.1038/nrg2717 20212494

[DEV131060C70] Yang L., Bula D., Arroyo J. G. and Chen D. F. (2004). Preventing retinal detachment–associated photoreceptor cell loss in Bax-deficient mice. *Invest. Ophthalmol. Vis. Sci.* 45, 648-654. 10.1167/iovs.03-0827 14744910

[DEV131060C71] Yang Y., Yang F., Xiong Z., Yan Y., Wang X., Nishino M., Mirkovic D., Nguyen J., Wang H. and Yang X.-F. (2005). An N-terminal region of translationally controlled tumor protein is required for its antiapoptotic activity. *Oncogene* 24, 4778-4788. 10.1038/sj.onc.1208666 15870695 PMC3901995

[DEV131060C72] Yenofsky R., Bergmann I. and Brawerman G. (1982). Messenger RNA species partially in a repressed state in mouse sarcoma ascites cells. *Proc. Natl. Acad. Sci. USA* 79, 5876-5880. 10.1073/pnas.79.19.5876 6964392 PMC347013

[DEV131060C73] Yoon B. C., Jung H., Dwivedy A., O'Hare C. M., Zivraj K. H. and Holt C. E. (2012). Local translation of extranuclear lamin B promotes axon maintenance. *Cell* 148, 752-764. 10.1016/j.cell.2011.11.064 22341447 PMC3314965

[DEV131060C74] Zala D., Hinckelmann M.-V., Yu H., Lyra da Cunha M. M., Liot G., Cordelières F. P., Marco S. and Saudou F. (2013). Vesicular glycolysis provides on-board energy for fast axonal transport. *Cell* 152, 479-491. 10.1016/j.cell.2012.12.029 23374344

[DEV131060C75] Zhang D., Li F., Weidner D., Mnjoyan Z. H. and Fujise K. (2002). Physical and functional interaction between myeloid cell leukemia 1 protein (MCL1) and Fortilin. The potential role of MCL1 as a fortilin chaperone. *J. Biol. Chem.* 277, 37430-37438. 10.1074/jbc.M207413200 12149273

[DEV131060C76] Zhang M., Pritchard M. R., Middleton F. A., Horton J. A. and Damron T. A. (2008). Microarray analysis of perichondral and reserve growth plate zones identifies differential gene expressions and signal pathways. *Bone* 43, 511-520. 10.1016/j.bone.2008.04.021 18579462 PMC2569855

[DEV131060C77] Zhang J., de Toledo S. M., Pandey B. N., Guo G., Pain D., Li H. and Azzam E. I. (2012). Role of the translationally controlled tumor protein in DNA damage sensing and repair. *Proc. Natl. Acad. Sci. USA* 109, E926-E933. 10.1073/pnas.1106300109 22451927 PMC3341051

[DEV131060C78] Zivraj K. H., Tung Y. C. L., Piper M., Gumy L., Fawcett J. W., Yeo G. S. and Holt C. E. (2010). Subcellular profiling reveals distinct and developmentally regulated repertoire of growth cone mRNAs. *J. Neurosci.* 30, 15464-15478. 10.1523/JNEUROSCI.1800-10.2010 21084603 PMC3683943

